# Effect of X-Ray Attenuation of Arterial Obstructions on Intravenous Thrombolysis and Outcome after Ischemic Stroke

**DOI:** 10.1371/journal.pone.0145683

**Published:** 2015-12-23

**Authors:** Grant Mair, Rüdiger von Kummer, Richard I. Lindley, Peter A. G. Sandercock, Joanna M. Wardlaw, IST-3 Collaborative Group

**Affiliations:** 1 Division of Neuroimaging Sciences, University of Edinburgh, Western General Hospital, Edinburgh, United Kingdom; 2 Department of Neuroradiology, Dresden University Stroke Centre, University Hospital, Dresden, Germany; 3 Westmead Hospital Clinical School and The George Institute for Global Health, University of Sydney, Sydney, Australia; 4 Division of Clinical Neurosciences, University of Edinburgh, Western General Hospital, Edinburgh, United Kingdom; National Cerebral and Cardiovascular Center, JAPAN

## Abstract

**Objective:**

To assess whether the x-ray attenuation of intra-arterial obstruction measured on non-contrast CT in ischemic stroke can predict response to thrombolysis and subsequent functional outcome.

**Methods:**

The Third International Stroke Trial (IST-3) was a multicenter randomized-controlled trial of intravenous thrombolysis (rt-PA) given within six hours of ischemic stroke. Ethical approval and informed consent were obtained. In a subgroup of 109 IST-3 patients (38 men, median age 82 years), a single reader, masked to all clinical and other imaging data, manually measured x-ray attenuation (Hounsfield Units, HU) on non-contrast CT at the location of angiographically-proven intra-arterial obstructions, pre-randomization and at 24–48 hour follow-up. We calculated change in attenuation between scans. We assessed the impact of pre-randomization arterial obstruction attenuation on six-month functional outcome.

**Results:**

Most arterial obstructions (64/109, 59%) were hyperattenuating (mean 51.0 HU). Compared with control, treatment with rt-PA was associated with a greater, but non-significant, reduction in obstruction attenuation at follow-up (-8.0 HU versus -1.4 HU in patients allocated control, p = 0.117). In multivariable ordinal regression analysis controlled for patient age, stroke severity, location and extent of obstruction, time from stroke onset to baseline scan and rt-PA treatment allocation, the attenuation of pre-randomization arterial obstruction was not independently associated with six-month outcome (odds ratio = 0.99, 95% confidence interval = 0.94–1.03, p = 0.516).

**Conclusions:**

In ischemic stroke, the x-ray attenuation of the arterial obstruction may decline more rapidly from baseline to 24–48 hours following treatment with thrombolysis but we found no evidence that baseline arterial obstruction attenuation predicts six-month outcome.

## Introduction

Histological analysis of intra-arterial obstructions such as thrombus or embolus extracted from ischemic stroke patients has suggested that obstructions with a greater red blood cell composition have a higher x-ray attenuation on CT (appear more white, are more dense) than obstructions which are predominantly fibrin based or of a mixed composition [1–3]. It has been postulated that the response to thrombolytic therapy may therefore be different among stroke patients with intra-arterial obstructions of differing x-ray attenuations. A few cohort studies have suggested that measurement of intra-arterial x-ray attenuation at the location of an obstruction on baseline non-contrast CT may help to predict recanalization following treatment with intravenous thrombolysis in stroke [4–6]. There has, however, been very little assessment of whether the improved rate of recanalization translates to an improvement in functional outcome following stroke [7,8]. To our knowledge, the importance of x-ray attenuation of arterial obstructions for predicting outcome has never been assessed in a randomized-controlled trial testing intravenous thrombolysis.

The Third International Stroke Trial (IST-3) was a large multi-center, randomized-controlled trial which tested intravenous thrombolysis (recombinant tissue plasminogen activator, rt-PA) given within 6 hours of ischemic stroke [9]. Baseline and follow-up brain imaging was performed for all IST-3 patients. In some centers, CT or MR angiography was also routinely obtained pre-randomization [10].

In this post-hoc subgroup exploration of IST-3 trial data we wished to investigate, in patients with ischemic stroke and an angiographically-proven intra-arterial obstruction at baseline, whether the x-ray attenuation of the obstruction is predictive of six-month functional outcome. We also aimed to measure short-term changes in the attenuation of arterial obstruction following treatment with intravenous rt-PA.

## Materials and Methods

### The Third International Stroke Trial

IST-3 was an international, multi-center, prospective, randomized, open, blinded endpoint (PROBE) trial of intravenous rt-PA in ischemic stroke. Ethical approval for IST-3 was granted by the Multicentre Research Ethics Committees, Scotland (MREC/99/0/78) and by local ethics committees. Research was undertaken in accordance with the Declaration of Helsinki. Patient enrolment, data collection, image analysis and adherence to CONSORT recommendations have been previously described [9,11]. Briefly, patients with acute stroke of any severity, with no upper age limit, were eligible for inclusion in the trial if intravenous rt-PA (alteplase) could be started within 6 hours of stroke onset and CT/MR imaging had excluded both intracranial hemorrhage and any structural stroke mimic. Written informed consent was obtained for all patients. Stroke severity prior to randomization was assessed with the National Institutes of Health Stroke Scale (NIHSS). Clinical stroke syndrome at baseline was assessed with the Oxfordshire Community Stroke Project (OCSP) classification [12]. After entry of baseline data, patients were randomized to receive intravenous rt-PA (0.9mg/Kg) or control. Patients were followed by postal questionnaire or telephone interview at six-months and functional status assessed with the Oxford Handicap Scale (OHS). IST-3 is registered with the *Current Controlled Trials* database, ISRCTN25765518, http://www.controlled-trials.com/ISRCTN25765518.

The imaging protocol required that non-contrast CT scans extend from the foramen magnum to vertex, with maximum slice thickness 4–5 mm through the posterior fossa and 8–10 mm for the cerebral hemispheres. There was no pre-defined requirement for thin-slice sections and all acquired data including spiral volumes were accepted. CT or MR angiography (CTA and MRA, respectively) were also collected if available; the published protocol for the IST-3 angiography substudy specified minimum acquisition standards [10]. Only IST-3 patients who had CTA or MRA performed within 2 hours of baseline non-contrast CT are included in this present analysis. We recorded time from stroke onset to baseline scan. Follow-up non-contrast CT imaging was also performed for all patients at 24–48 hours; follow-up delay was also recorded. Follow-up angiography was not mandatory in the IST-3 angiography substudy [10].

### Image analysis

A single neuroradiologist independently evaluated all baseline angiography in IST-3, blind to treatment allocation, clinical details and outcome, to identify patients for subgroup analysis, i.e. those with a filling defect on angiography that could be assessed on the corresponding non-contrast CT. Previous IST-3 scan reading panel assessments were not used for this report. Image analysis was also performed blind to any subsequent imaging or other scan reads. Image evaluation was performed using standard DICOM software (Digital Jacket, DesAcc, Bellevue, WA, USA). Results were entered into a database using a pre-validated structured proforma (available at: http://www.bric.ed.ac.uk/research/imageanalysis.html).

Angiography was assessed using the IST-3 angiography score [10]: 0 = occluded, 1 = minimal patency (some contrast penetrates obstruction but none or only a minimal amount enters distal artery), 2 = patency <50% of the lumen at the point of obstruction and a) only partly filling (<half) or b) incomplete filling (but >half) of the major branches of the affected artery, 3 = patency >50% of the lumen and filling of most of the branches of the affected artery, 4 = normal. We have previously demonstrated inter- and intra-observer reliability for the IST-3 angiography score in the assessment of CTA; inter-observer agreement was substantial for both (Krippendorff’s Alpha = 0.66 and 0.63, respectively) [13]. We stratified angiographic obstruction by the three largest intracranial arterial segments involved: internal carotid artery (ICA), main stem of middle cerebral artery (MCA), sylvian branch of MCA, anterior cerebral artery (ACA), posterior cerebral artery (PCA), vertebral artery (VA) and the basilar artery (BA). To assess the extent of ischemia on baseline non-contrast CT we calculated ASPECTS (Alberta Stroke Program Early CT Score) for all patients [14].

**Fig 1:**
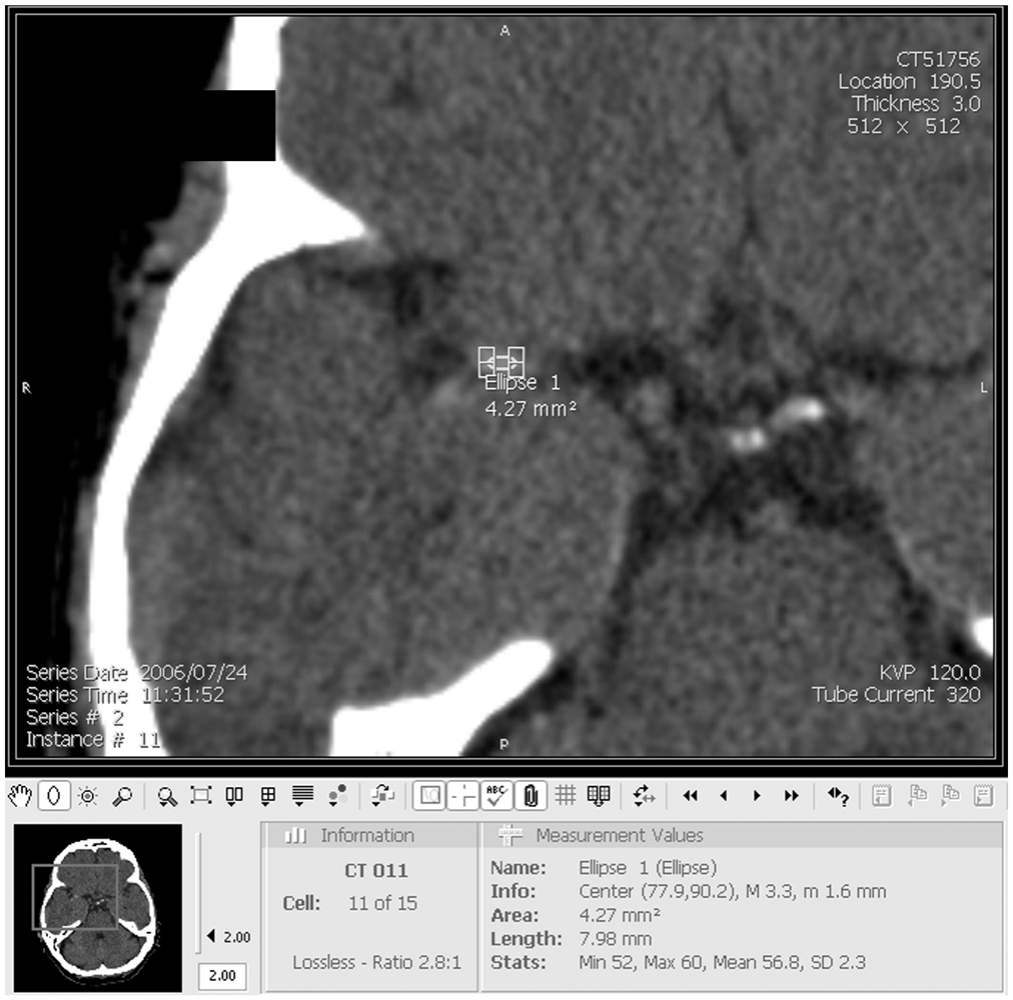
Intra-arterial HU measurement technique. For each intra-arterial measurement location (level of obstruction, contralateral normal vessel, basilar artery), 3 elliptical regions of interest were applied by hand on a magnified image. Note that *Ellipse 1* is placed within the horizontal segment of the right middle cerebral artery. HU = Hounsfield Unit.

Intra-arterial x-ray attenuation was measured on the non-contrast CT with reference to the concurrent abnormal CTA/MRA. Simultaneous parallel viewing of the non-contrast CT and the CTA/MRA allowed for attenuation measures (Hounsfield Units, HU) to be taken from within the same location on non-contrast CT that corresponded with the filling defect on angiography (i.e. the obstruction). Patients were excluded from the study if the obstruction was insufficient to allow for attenuation measures to be performed (i.e. minor stenosis without a discrete measurable filling defect). In addition to analysing the obstruction, we also obtained attenuation measures within the equivalent contralateral normal artery if possible (for example, the opposite MCA at an equivalent location) and also within the basilar artery. We therefore measured intra-arterial attenuation at three intra-cranial locations for most patients (one obstructed artery and two normal controls). At each location, three separate elliptical regions of interest were applied by hand, to include as much of the intra-vascular area as possible but taking care not to include the arterial wall or any surrounding tissue; a mean attenuation value of the three readings was later calculated. Attenuation measurements were performed following x2 magnification of images (Fig 1). The subjective appearance of arterial obstruction on baseline non-contrast CT was also noted as either isoattenuating to normal vessels or hyperattenuating (if obstruction appeared brighter than adjacent or equivalent contralateral arteries but non-calcified [15]). We measured the length of hyperattenuated arterial obstructions on non-contrast CT. We calculated HU ratios for each patient to assess attenuation of the obstruction in relation to normal background intra-arterial attenuation as: mean obstruction HU ÷ mean normal vessel HU. We repeated obstruction attenuation measurements, in the same location, on follow-up non-contrast CT. We calculated change in attenuation of the obstruction between the two scans.

### Statistical analysis

We used t-tests and Mann-Whitney U-tests to compare characteristics of the subgroup and the full IST-3 population and to compare attenuation values, as appropriate. We used Pearson’s correlation coefficient, analysis of variance and t-tests to look for associations between baseline obstruction HU and clinical characteristics, other imaging appearances and outcome in separate univariate analyses. Similarly and as previously described [9,16,17], we used adjusted multivariable ordinal regression analysis (adjusted for patient age, time from stroke onset to baseline scan, NIHSS, location of arterial obstruction, extent of arterial obstruction, IST-3 angiography score and treatment allocation) to look for independent associations between baseline attenuation of obstruction and outcome. Two adjusted regression analyses were performed: the first included attenuation of obstruction as a continuous variable (i.e. absolute HU, quantitative); the second included attenuation as a dichotomous variable (i.e. iso- or hyper-attenuating, qualitative).

All analyses were performed using IBM SPSS Statistics software, version 20.0 (IBM Corporation, Armonk, NY, USA). We considered a p-value <0.05 significant.

## Results

### IST-3 subgroup

In IST-3, 109 patients with baseline angiography (108 CTA and 1 MRA) had both a concurrent non-contrast CT and a measurable angiographic stenosis or occlusion. Compared with the whole IST-3 trial population (n = 3035), this subgroup had significantly more severe strokes at baseline (median NIHSS 17 versus 11 for the whole trial, p<0.001), a different distribution of clinical stroke syndromes (e.g. more total anterior circulation infarcts, 62.4% versus 42.3% for the whole trial, and fewer lacunar infarcts, 3.7% versus 11.2% for the whole trial, p<0.001), more extensive ischemia visible on baseline non-contrast CT (median ASPECTS 9 versus 10 for the whole trial, p = 0.001) and a worse six-month functional outcome (median OHS 5 versus 4 for the whole trial, p<0.001). In addition, there were significantly fewer men in the subgroup (35% versus 48% in the whole trial, p = 0.004). There were no significant differences in patient age (median 82 years in subgroup), presence of baseline atrial fibrillation (n = 35, 32.1% of subgroup), percentage treated with rt-PA (n = 51, 46.8% of subgroup), time from stroke onset to baseline scan (mean 171 minutes in subgroup), time to randomization (mean 230 minutes in subgroup) or time to imaging follow-up (median 25 hours in subgroup) between the subgroup and the whole IST-3 trial, S1 Table.

### Imaging findings

Table 1 demonstrates the results of imaging assessment for the 109 patients. We identified a hyperattenuating artery in 64 patients (59%). Within these hyperattenuating arterial obstructions, the mean was 51.0 HU. This compares with a mean of 37.9 HU in isoattenuating obstructions, p<0.001. The mean within normal vessels (i.e. normally flowing blood) was 38.1 HU. The mean HU ratio of hyperattenuating obstructions was 1.38 compared with a mean HU ratio of 0.97 for isoattenuating obstructions, p<0.001. The majority of patients had obstructions within the ICA (20%) or MCA (75%). In most patients, obstruction was isolated to a single arterial segment (56%), fewer patients had obstruction affecting 2 or 3 arterial segments (37% and 7%, respectively). The mean length of hyperattenuating arterial segment on non-contrast CT was 19 mm (standard deviation 10 mm). By IST-3 angiography score, most patients had >50% luminal stenosis (84%) and in 44%, there was either complete occlusion or severe luminal stenosis.

**Table 1:** Imaging characteristics of the IST-3 subgroup with measurable intra-arterial obstruction (n = 109).

**A**	
**Intra-arterial X-ray attenuation on non-contrast CT**	**Mean (SD)**
HU of *hyperattenuating* obstruction at baseline (mean, SD)	51.0 (8.3)
HU of *isoattenuating* obstruction at baseline (mean, SD)	37.9 (10.7)
HU of non-obstructed vessel (mean, SD)	38.1 (5.2)
HU at follow-up of obstructions *hyperattenuating* at baseline (mean, SD)	45.1 (9.2)
HU at follow-up of obstructions *isoattenuating* at baseline (mean, SD)	38.2 (8.2)
Ratio of obstruction: normal vessel for obstructions *hyperattenuating* at baseline (mean, SD)	1.38 (0.23)
Ratio of obstruction: normal vessel HU for obstructions *isoattenuating* at baseline (mean, SD)	0.97 (0.21)
**B**	
**Angiographic location of arterial obstruction**	**n (%)**
ICA	22 (20.2)
MCA mainstem	61 (55.9)
MCA sylvian branch	21 (19.3)
ACA	0
PCA	0
Vertebral artery	2 (1.8)
Basilar artery	3 (2.8)
**C**	
**Number of obstructed arterial segments on angiography**	**n (%)**
1	61 (56.0)
2	40 (36.7)
3	8 (7.3)
**D**	
**IST-3 Angiography Score** ^a^	**n (%)**
0	11 (10.1)
1	37 (33.9)
2a	17 (15.6)
2b	27 (24.8)
3	17 (15.6)
4	0

HU = Hounsfield Units. SD = Standard Deviation. ICA = Internal Carotid Artery. MCA = Middle Cerebral Artery. ACA = Anterior Cerebral Artery. PCA = Posterior Cerebral Artery.

^a^IST-3 angiography scoring: 0 = occluded, 1 = minimal patency (some contrast penetrates obstruction but no/minimal enters distal artery), 2 = patency <50% of the lumen at the point of obstruction and a) only partly filling (<half) or b) incomplete filling (but >half) of the major branches of the affected artery, 3 = patency >50% of the lumen and filling of most of the branches of the affected artery, 4 = normal artery.

### Associations with obstruction attenuation at baseline

On univariate analysis, there was no significant difference in the mean attenuation of baseline arterial obstruction when patients with versus without atrial fibrillation were compared (45.9 HU versus 45.5 HU, respectively, p = 0.859) or among the different clinical stroke subtypes (F = 1.5, p = 0.220). Similar non-significant results were obtained in all cases if those with hyper- versus iso-attenuating arterial obstructions were compared. There was no correlation between the length of the hyperattenuating arterial segment and the attenuation of that obstruction (r = 0.03, p = 0.825). Similarly, the mean attenuation of arterial obstruction at baseline did not differ between those with 1 versus those with 2 or 3 obstructed arterial segments (44.8 HU versus 46.6 HU, respectively, p = 0.418). Finally, there was no correlation between the time from stroke onset to baseline scan and the attenuation of arterial obstruction on the baseline scan (r = 0.13, p = 0.203).

### Effect of rt-PA on obstruction attenuation

**Fig 2:**
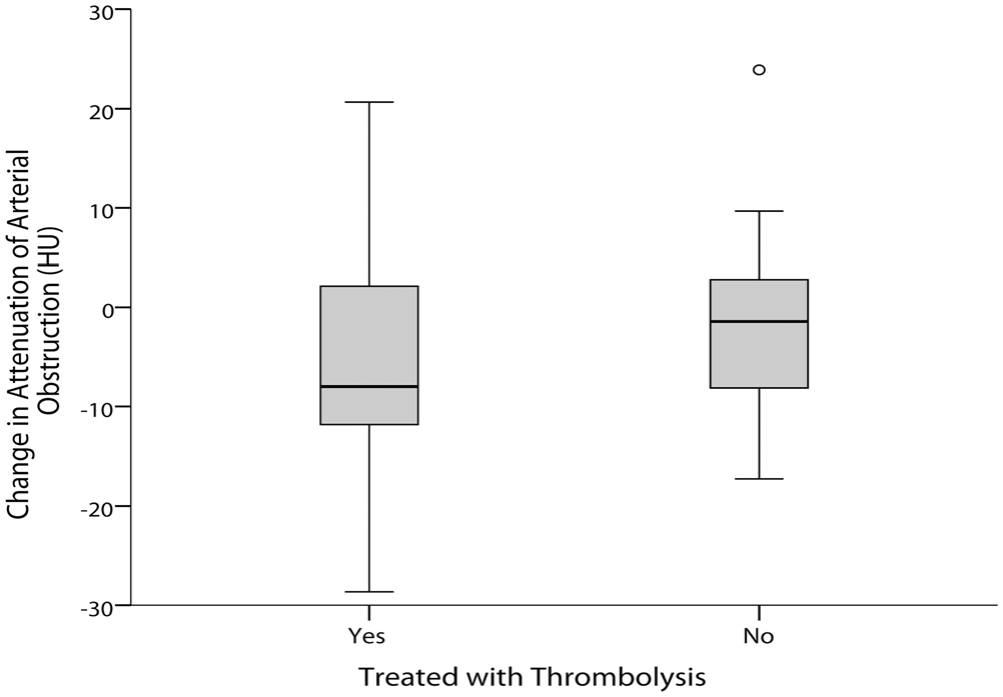
Effect of thrombolysis on change in attenuation of arterial obstruction between baseline and follow-up CT. A negative change in arterial obstruction HU (Hounsfield Units) between baseline and follow-up CT indicates a reduction in attenuation of the obstruction.

The median time between baseline and follow-up non-contrast CT scans was 25 hours. At follow-up, hyperattenuating obstructions had reduced to a mean of 45.1 HU while isoattenuating obstructions were unchanged at mean 38.2 HU. There was a non-significantly (p = 0.117) greater reduction of attenuation between baseline and follow-up non-contrast CT for arterial obstructions in patients treated with rt-PA (median reduction of 8.0 HU) versus controls (median reduction of 1.4 HU), Fig 2. Similar results were obtained if only patients with hyperattenuating obstructions at baseline were included; median reduction of 8.4 HU in those treated with rt-PA versus a median reduction of 0.5 HU in those allocated control, p = 0.094.

Analysis includes patients with both iso- and hyper-attenuating obstructions at baseline.

There was no significant difference between the groups treated with thrombolysis (median -8.0 HU, IQR -12.4 to 2.5) and control (median -1.4 HU, IQR -8.7 to 2.9), p = 0.117.

### Obstruction attenuation and six-month outcome

There were no significant associations on univariate analysis between the attenuation of arterial obstruction at baseline and six-month functional outcome in either the rt-PA or control groups. For correlation between baseline obstruction attenuation and six-month OHS the results were: r = 0.03, p = 0.851 for those treated with rt-PA (n = 51) and r = 0.13, p = 0.342 in the control group (n = 58). Comparing baseline arterial obstruction attenuation between the patients reaching independence (OHS 0–2) and those patients dependent or dead at six months (OHS 3–6), the mean attenuation difference was: 0.2 HU, p = 0.944 for those treated with rt-PA and 1.5 HU, p = 0.673 in the control group. In both cases, baseline HU values were non-significantly higher among patients with poor six-month functional outcome (OHS 3–6).

In the main IST-3 trial (n = 3035), in an adjusted ordinal regression analysis, rt-PA treatment was associated with a favourable shift in OHS (better outcomes) at six months (common odds ratio, OR = 1.27, 95%CI = 1.10–1.47; p = 0.001) [9]. Among subgroup patients (n = 109), our estimates of rt-PA effect on OHS derived using similar adjusted ordinal regression analyses were not materially different (e.g. OR = 2.17, 95%CI = 0.80–5.91) to the main IST-3 trial results but were less precise because of the small sample size. We additionally found no evidence that baseline arterial obstruction attenuation was an independent predictor of outcome in either the quantitative (absolute HU of obstruction, OR = 0.99, 95%CI = 0.94–1.03, p = 0.516) or qualitative analyses (hyper- versus iso-attenuating obstruction, OR = 0.53, 95%CI = 0.20–1.41, p = 0.203), Tables 2 and 3 respectively. There was a non-significant trend towards poorer outcome among patients with hyperattenuating rather than isoattenuating obstructions.

**Table 2:** Ordinal regression analysis with six-month OHS as the dependant variable (n = 109); attenuation of obstruction is included as a quantitative continuous variable.

	Odds Ratio^a^	95% Confidence Interval	p-value
Age (years)	0.95	0.92–0.98	0.001
Time from stroke onset to baseline scan (hours)	1.12	0.78–1.62	0.539
NIHSS	0.85	0.79–0.92	<0.0001
Baseline attenuation (HU) of arterial obstruction	0.99	0.94–1.03	0.516
Location of arterial obstruction	1.10	0.73–1.68	0.645
Number of obstructed arterial segments (1–3)	0.70	0.30–1.62	0.407
IST-3 angiography score (0–3)	1.03	0.71–1.51	0.865
Treated with rt-PA (versus control)	2.17	0.80–5.91	0.131

NIHSS = National Institutes of Health Stroke Scale. HU = Hounsfield Units. rt-PA = recombinant tissue plasminogen activator.

^a^Odds ratio <1 indicates a worse outcome.

**Table 3:** Ordinal regression analysis with six-month OHS as the dependant variable (n = 109); attenuation of obstruction is included as a qualitative dichotomous variable.

	Odds Ratio^a^	95% Confidence Interval	p-value
Age (years)	0.95	0.92–0.98	0.001
Time from stroke onset to baseline scan (hours)	1.13	0.79–1.63	0.508
NIHSS	0.86	0.79–0.92	<0.0001
Hyperattenuating obstruction at baseline (versus isoattenuating obstruction)	0.53	0.20–1.41	0.203
Location of arterial obstruction	1.12	0.75–1.67	0.589
Number of obstructed arterial segments (1–3)	0.70	0.30–1.60	0.396
IST-3 angiography score (0–3)	1.02	0.70–1.49	0.902
Treated with rt-PA (versus control)	2.29	0.85–6.17	0.102

NIHSS = National Institutes of Health Stroke Scale. rt-PA = recombinant tissue plasminogen activator.

^a^Odds ratio <1 indicates a worse outcome.

For all analyses, replacing absolute arterial obstruction attenuation values with obstruction attenuation ratios did not alter the results (data not shown).

## Discussion

These data from an angiographic substudy within the largest randomized controlled-trial testing intravenous rt-PA in ischemic stroke do not support the hypothesis that baseline x-ray attenuation of intra-arterial obstruction measured on non-contrast CT is an independent predictor of functional outcome at 6 months. Since age, baseline stroke severity and time to treatment are such powerful predictors of outcome after ischemic stroke treated with intravenous thrombolysis, it is perhaps not surprising that we have been unable to show that measuring the attenuation of arterial obstruction adds predictive value for decisions about intravenous rt-PA treatment.

The value of measuring arterial obstruction attenuation on CT to select patients for treatment is conflicting. Two non-randomized studies (both around 100 patients) found no association between the attenuation of arterial obstruction and outcome after thrombolysis [7,8]. Three smaller cohort studies have however demonstrated with highly consistent results that intravenous thrombolysis more often resulted in successful arterial recanalization in patients with highly attenuating obstructions at baseline. Puig et. al. (n = 45) demonstrated a mean relative HU (equivalent to mean HU ratio in our study) of 1.57 for patients achieving recanalization versus 1.11 for those without recanalization (p<0.001) [4]. Similarly, Moftakhar et. al. (n = 90) demonstrated a mean relative HU of 1.58 in patients that recanalized versus 1.39 in those that did not (p = 0.01) [5]. Finally, Niesten et. al. (n = 88) demonstrated a mean relative HU of 1.54 in patients that recanalized versus 1.29 in those that did not (p<0.001) [6]. We unable to assess recanalization following treatment with intravenous rt-PA as follow-up angiography was not mandatory in IST-3. The apparent discrepancy between our results and these three cohort studies may indicate that success of recanalization therapy is not the only predictor of outcome. Indeed the literature assessing relationships between the attenuation of obstruction and success of endovascular thrombus retrieval, has demonstrated that while recanalization is more likely with hyperattenuating obstructions, where reported, this does not translate to a proportionally improved functional outcome [18–22]. In addition, some authors have identified significant differences in arterial obstruction attenuation when stroke cohorts are stratified by etiological stroke subtype. For example, cardioembolic thrombus may be more attenuating than atherothrombotic thrombus [4,23], but such results have not always been replicated [8] and a large retrospective analysis of over 8000 patients failed to demonstrate an independent association between hyperattenuating thrombus and stroke etiology [24]. We similarly did not find any association in IST-3 between clinical stroke subtype (which may vary with differing underlying etiologies [12]) or the presence of atrial fibrillation and the attenuation of arterial obstruction.

Our secondary analysis assessing the change in attenuation of arterial obstruction from baseline to short-term follow-up CT is consistent with the hypothesis that the HU of obstruction may reduce more rapidly in patients given intravenous rt-PA than controls; however, the observed difference in attenuation was non-significant. This result is also consistent with IST-3 data showing that intravenous thrombolysis accelerates the removal of the hyperattenuating artery sign (evident at baseline in over 700 patients), a recognised surrogate for intra-arterial thrombus [17].

Our study has limitations. In a large pragmatic multicenter trial like IST-3 there is inevitably some variability in scan parameters and protocols but, nevertheless, the study design closely represents normal working practice. We cannot however exclude the possibility that x-ray attenuation of arterial obstructions was under-estimated due to partial volume effects of thick CT slices for some patients. Similarly, partial volume effects may have reduced the accuracy of attenuation measurements in some cases. IST-3 angiography was performed only in some centers and may therefore be biased toward patients for whom it could convey the greatest perceived benefit. Though our total patient numbers for this assessment are similar to previous work, our analyses are underpowered. But as a randomized-controlled trial, IST-3 provides an unbiased assessment of the relationships between the use of intravenous rt-PA and functional outcome and our estimate of the rt-PA effect on outcome in this subgroup is not substantially different from that reported in the full IST-3 trial. A single reader manually applied regions of interest to measure intra-arterial attenuation in our study. Repeat assessment by a secondary reader or use of an automated segmentation program may have improved the accuracy of the data collected. We used standardized and pre-validated methods of accessing and scoring imaging with blinding of the reader to allow for robust and repeatable data collection.

## Conclusions

These data from a prospective analysis of a randomized-controlled trial do not provide evidence that measurement of the x-ray attenuation of arterial obstruction on CT adds useful additional information on prognosis or response to intravenous thrombolysis in patients with ischemic stroke.

## Supporting Information

S1 Table Clinical characteristics of the full IST-3 trial population and the subgroup with measurable intra-arterial obstruction on angiography. (DOCX)
