# Supplementary material for: Effect of X-Ray Attenuation of Arterial Obstructions on Intravenous Thrombolysis and Outcome after Ischemic Stroke
Source: PLoS One. 2015 Dec 23;10(12):e0145683. doi: 10.1371/journal.pone.0145683 (PMC4689396; doi:10.1371/journal.pone.0145683)
Supplement: S1 Table — (DOCX) [file pone.0145683.s001.docx]

**SUPPORTING INFORMATION**

**Effect of X-Ray Attenuation of Arterial Obstructions on Intravenous Thrombolysis and Outcome after Ischemic Stroke**

Grant Mair^1^, Rüdiger von Kummer^2^, Richard I Lindley^3^, Peter AG Sandercock^4^, Joanna M Wardlaw*^1^, on behalf of the IST-3 Collaborative Group^

1. Division of Neuroimaging Sciences, University of Edinburgh, Western General Hospital, Edinburgh, UK

2. Department of Neuroradiology, Dresden University Stroke Centre, University Hospital, Dresden, Germany

3. Westmead Hospital Clinical School and The George Institute for Global Health, University of Sydney, Australia

4. Division of Clinical Neurosciences, University of Edinburgh, Western General Hospital, Edinburgh, UK

* Corresponding author

Email: [joanna.wardlaw@ed.ac.uk](mailto:joanna.wardlaw@ed.ac.uk) (JMW)

^ Members of the IST-3 Collaborative Group are listed in Acknowledgements

**S1 Table.** Clinical characteristics of the full IST-3 trial population and the subgroup with measurable intra-arterial obstruction

|  | **Arterial Obstruction Subgroup (n=109)** | **Full IST-3 Group**  **(n=2926)** | **p-value for Difference*** |
| --- | --- | --- | --- |
| **Age** (median, IQR) | 82 (75-86) years | 81 (72-86) years | 0.192 |
| **Male Sex** (n, %) | 38 (34.9) | 1465 (48.3) | 0.004 |
| **Clinical stroke syndrome** (n, %)  TACI  PACI  POCI  LACI  Other | 68 (62.4)  32 (29.4)  5 (4.6)  4 (3.7)  0 | 1238 (42.3)  1114 (38.1)  241 (8.2)  328 (11.2)  5 (0.2) | <0.001 |
| **Atrial fibrillation at baseline** | 35 (32.1) | 879 (30.0) | 0.644 |
| **Time from stroke onset to baseline CT** (mean, SD) | 171 (79) minutes | 164 (73) minutes | 0.282 |
| **Time from stroke onset to randomization** (mean, SD) | 230 (79) minutes | 231 (73) minutes | 0.891 |
| **Baseline NIHSS** (median, IQR) | 17 (11-21) | 11 (6-17) | <0.001 |
| **Baseline ASPECTS** (median, IQR) | 9 (6-10) | 10 (8-10) | 0.001 |
| **Treated with rt-PA** (n, %) | 51 (46.8) | 1515 (49.9) | 0.506 |
| **Time from baseline to follow-up CT** (median, IQR) | 25 (24-29) hours | 26 (24-36) hours | 0.110 |
| **Six-month OHS** (median, IQR) | 5 (3-6) | 4 (2-6) | <0.001 |
| **Independent at six-months** (n OHS 0-2, %) | 26 (23.9) | 1088 (35.8) | 0.008 |
| **Dead by six-months** (n, %) | 43 (39.4) | 815 (26.9) | 0.003 |

TACI = Total Anterior Circulation Infarct. PACI = Partial Anterior Circulation Infarct. POCI = Posterior Circulation Infarct. LACI = Lacunar Infarct. NIHSS = National Institutes of Health Stroke Scale. ASPECTS = Alberta Stroke Program Early CT Score. OHS = Oxford Handicap Scale. IQR = Interquartile Range. SD = Standard Deviation.

* The arterial obstruction attenuation subgroup was compared with the remainder of the full IST-3 group, i.e. 3035 minus 109 = 2926
